# Acute Left Bundle Branch Injury During Deep Septal Lead Implantation

**DOI:** 10.19102/icrm.2023.14053

**Published:** 2023-05-15

**Authors:** Favour Markson, Richard Orji, Hakeem Ayinde

**Affiliations:** ^1^Department of Medicine, Lincoln Medical Center, New York, NY, USA; ^2^Department of Medicine, Rosalind Franklin University, North Chicago, IL, USA; ^3^Department of Medicine, Northwestern Medicine Hospital, McHenry, IL, USA; ^4^Division of Cardiology, Cardiology Associates of Fredericksburg, Fredericksburg, VA, USA

**Keywords:** Conduction abnormalities, conduction system pacing, left bundle branch block, left bundle branch injury, left bundle branch pacing

## Abstract

Left bundle branch pacing (LBBP) is a novel technique that has emerged as an alternative method for conduction system pacing. As a new modality, this procedure may carry complications that are yet to be explored. This report describes a case of injury to the left bundle branch during deep septal lead implantation for LBBP.

## Introduction

Cardiac pacing is an effective treatment for various arrhythmias, which arise due to abnormalities of the intrinsic pacemaker or conducting system of the heart.^1^ Right ventricular apical pacing has been the standard therapy for cardiac pacing; however, due to its limitations, we have witnessed the emergence of conduction system pacing techniques, His-bundle pacing (HBP), and left bundle branch (LBB) pacing (LBBP). LBBP captures the proximal left bundle, its branches, and the left ventricular septal myocardium through a transventricular septal approach at a lower threshold, making this technique a superior alternative to HBP.^2^ Similarly, it overcomes electrical and mechanical dyssynchrony, which are significant limitations of right ventricular apical pacing.^3^ Recent studies have demonstrated a high implant success rate with promising safety outcomes of LBBP.^1,3^ However, as a novel technique, some inherent complications related to the procedure have not yet been well documented.

In this report, we describe a case of acute LBB injury following septal lead implantation, an unreported complication of LBBP.

## Case presentation

A 92-year-old woman was brought to the emergency department following a syncopal episode at rest. She had reported prior episodes of dizziness 1 day before the syncopal attack.

On her arrival to emergency medical services, her heart rate was 30 bpm in sinus rhythm, her blood pressure was 136/70 mmHg, she weighed 70 kg, and her body mass index was 25 kg/m^2^. Her physical examination and laboratory studies were unremarkable. Her personal history was notable for paroxysmal atrial fibrillation controlled with 50 mg of flecainide twice daily and 50 mg of metoprolol tartrate twice daily.

**Figure 1:**
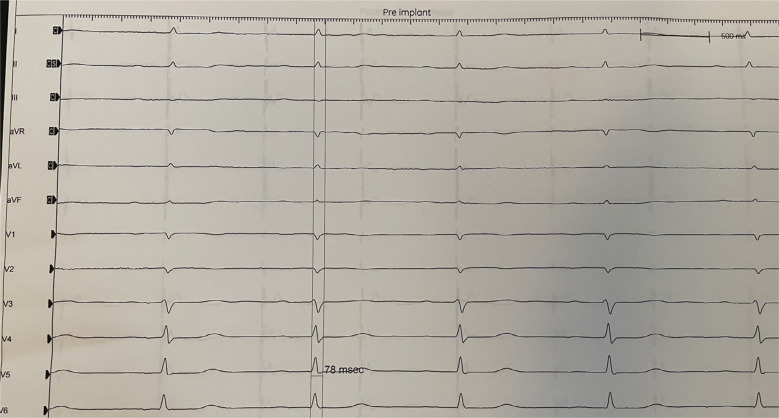
Electrocardiogram on hospital admission showing a normal QRS morphology with bradycardia (58 bpm).

A baseline electrocardiogram revealed sinus bradycardia at a rate of 58 bpm and a normal QRS duration **(Figure 1)**. An echocardiogram showed a normal left ventricular size and systolic function. The left ventricular septal diameter was 0.8 cm, and the left ventricular internal diastolic diameter was 4.3 cm.

**Figure 2:**
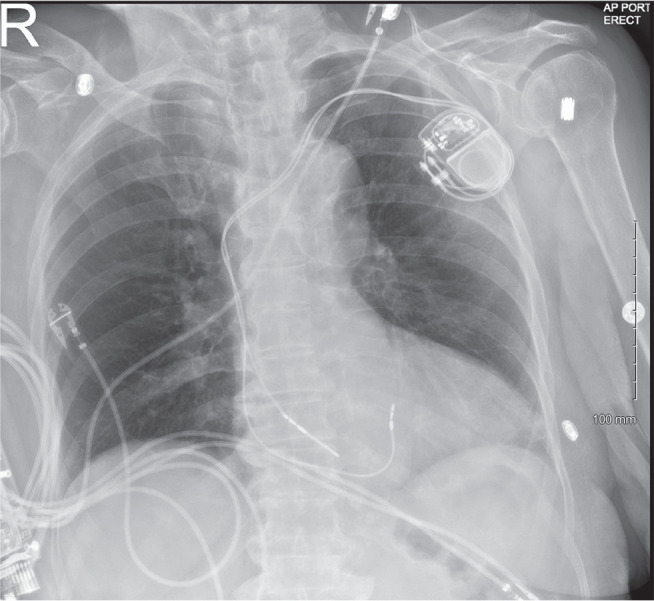
Chest X-ray showing pacemaker with leads implanted in the right atrial appendage and deep into the interventricular septum.

She was diagnosed with cardiac syncope secondary to sinus node dysfunction. After a discussion of the risks and benefits, she agreed to proceed with the implantation of a dual-chamber pacemaker. Subsequently, a dual-chamber pacemaker (Biotronik, Berlin, Germany) was implanted, with the goal of LBB area pacing in the ventricle **(Figure 2)**.

**Figure 3:**
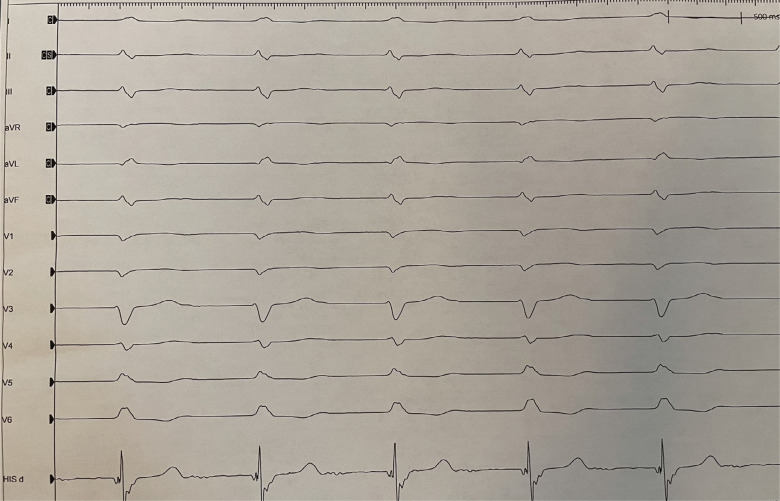
Electrocardiogram with left bundle branch injury currents noted during deep septal lead implantation.

**Figure 4:**
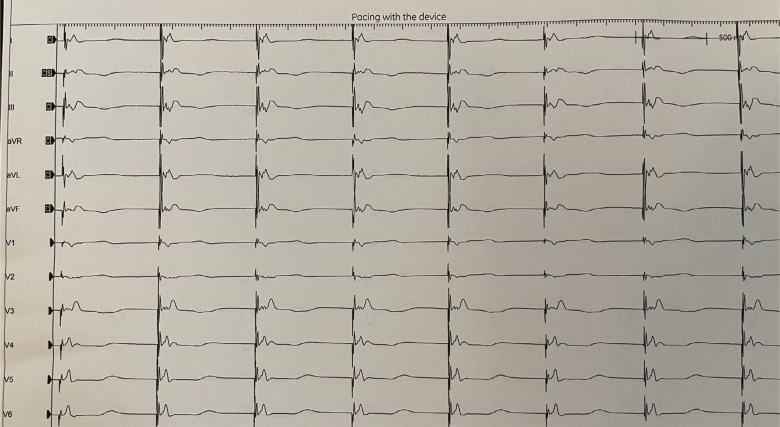
Recording of the paced QRS morphology of the left bundle branch, confirming capture with the right bundle branch pattern in the v1 lead (qR).

Using unipolar mapping, the LBB region was identified on the proximal right ventricular septum. The Solia S lead (Biotronik) was then actively fixed in the septum while monitoring for impedance and changes in QRS morphology. Interestingly, the patient developed a new left bundle branch block (LBBB) during lead fixation **(Figure 3)**, suggesting a traumatic implant to the left bundle. However, pacing revealed recruitment of the left bundle with a left ventricular activation time of 53 ms **(Figure 4)**. The bipolar sensing on the ventricular lead was 5.5 mV, the unipolar pacing threshold was 0.8 V at 0.4 ms, and the unipolar impedance was 351 Ω. The patient was observed overnight. The following day, her ventricular lead function was similar with bipolar sensing at 11.2 mV, a unipolar pacing threshold of 0.8 V at 0.4 ms, and unipolar impedance at 312 Ω. As such, the LBBB had recovered, and the patient was discharged in stable condition for follow-up in the device clinic.

## Discussion

This case highlights a rare complication of intraseptal lead implantation for LBBP. Intraseptal LBB area pacing is a novel technique applied to directly capture the conduction system, with the capability of recruiting the left bundle fibers beyond a site of block.

In contrast to HBP, where physiologic ventricular pacing occurs via lead implantation at the relatively thinner His-bundle region, LBBP captures the ventricles through a pacing lead positioned deep into the left ventricular septum along the path of the LBB.^4^ This allows for better lead stability and lower thresholds with LBBP.

Early experience with conduction system pacing has shown its high success and good safety profile. However, there are reported cases of lesions at various levels of the conducting pathway during lead implantation, which leads to iatrogenic heart block. A study by Vijayaraman et al. revealed that, in a cohort of 358 patients who underwent HBP, 7.8% of patients developed an acute form of conduction system injury, with cases of right bundle branch block (5.8%), LBBB (0.8%), and complete heart block (1.1%) documented, respectively.^5^ To the best of our knowledge, this is the first reported case of LBB injury during deep septal implantation for LBBP, and its incidence remains unknown.

The most likely mechanism of the observed LBBB pattern in our patient was a direct injury to the left bundle beyond its fibrous insulation, which was sustained during deep penetration into the interventricular septum. The damage to the LBBB is mostly transient, with complete recovery in minutes to hours, as was evident in our patient. Despite injury, the LBB fibers were still recruited at a low pacing threshold. The rapid resolution of acute LBBB signifies tissue edema around conducting tissues during lead implantation as the most likely reason for bundle injury. Similarly, this transient nature of BBB in lead implantation has been described following injury to the His bundle with pacing at high thresholds that subsequently improved to capturing at a low threshold by the end of the procedure after the tissue edema had resolved.^6,7^

With the majority of LBB injuries being transient, the coincidence of an LBBB during lead implantation should not influence the reposition of the implanted lead, as similar cases with His-bundle injury have shown an excellent benefit of capturing at a low threshold in subsequent follow-up.^8^ However, a complete atrioventricular block can develop in rare instances; therefore, precautionary measures should be taken to prevent prolonged asystole. First, adequate knowledge of the anatomy of the septal area, its thickness, and the arborization of the LBB is essential. Additionally, the operator should be prepared to provide backup pacing with a transcutaneous or transvenous pacer during the procedure, especially in situations where there is a baseline diseased right bundle branch or LBB. Other reported risks noted with LBBP include lead dislodgement, septal perforation, concomitant injury to the right bundle branch during manipulation in the basal septum, and trauma to the septal arteries.^1,3,9^

In conclusion, LBBP effectively captures the ventricles at low thresholds with a high success rate. However, it is essential to apply caution against complete heart block as there is a risk of injury to the conducting pathway during lead implantation. Investigation into the safety outcomes of LBB area pacing remains an area for further exploration.
